# Dual-encoded magnetization transfer and diffusion imaging and its application to tract-specific microstructure mapping

**DOI:** 10.1162/imag_a_00019

**Published:** 2023-09-26

**Authors:** Ilana R. Leppert, Pietro Bontempi, Christopher D. Rowley, Jennifer S.W. Campbell, Mark C. Nelson, Simona Schiavi, G. Bruce Pike, Alessandro Daducci, Christine L. Tardif

**Affiliations:** McConnell Brain Imaging Centre, Montreal Neurological institute and Hospital, Montreal, Canada; Department of Computer Science, University of Verona, Verona, Italy; Department of Neurology and Neurosurgery, McGill University, Montreal, Canada; Departments of Radiology and Clinical Neuroscience, Hotchkiss Brain Institute, University of Calgary, Calgary, Canada; Department of Biomedical Engineering, McGill University, Montreal, Canada

**Keywords:** dual-encoding, magnetization transfer, diffusion, myelin, microstructure, connectome

## Abstract

We present a novel dual-encoded magnetization transfer (MT) and diffusion-weighted sequence and demonstrate its potential to resolve distinct properties of white matter fiber tracts at the sub-voxel level. The sequence was designed and optimized for maximal MT ratio (MTR) efficiency. The resulting whole brain 2.6 mm isotropic protocol to measure tract-specific MTR has a scan time under 7 minutes. Ten healthy subjects were scanned twice to assess repeatability. Two different analysis methods were contrasted: a technique to extract tract-specific MTR using Convex Optimization Modeling for Microstructure Informed Tractography (COMMIT), a global optimization technique; and conventional MTR tractometry. The results demonstrate that the tract-specific method can reliably resolve the MT ratios of major white matter fiber pathways and is less affected by partial volume effects than conventional multi-modal tractometry. By reducing the contamination due to partial volume averaging of tracts, dual-encoded MT and diffusion may increase the sensitivity to microstructure alterations of specific tracts due to disease, aging, or learning, as well as lead to weighted structural connectomes with more anatomical specificity.

## 1 Introduction

Magnetic Resonance Imaging (MRI) offers valuable insight into the morphology, composition, and microstructural organization of white matter fiber pathways in the brain. Many MR contrasts are sensitive to tissue myelin content, including T_1_ and T_2_ relaxation times (review Does, 2018) and magnetization transfer (review Sled, 2018). These MR markers have been shown to correlate to varying degrees with myelin density derived from histology (see review in Lazari & Lipp, 2021; Mancini et al., 2020). Through the combination of multiple contrast mechanisms, complementary properties of the underlying microstructure can be probed (Callaghan et al., 2014; Kolind et al., 2008) and more specific microstructural indices estimated (see review in Cercignani & Bouyagoub, 2018). For instance, myelin imaging can be combined with diffusion imaging to estimate the thickness of the myelin sheath relative to axon caliber, known as the g-ratio, using a biophysical model (Stikov et al., 2015). Multiple myelin-sensitive contrasts can also be combined, for example, T_2_^*^ and magnetization transfer (Mangeat et al., 2015), to help minimize the impact of confounds, such as iron content or field non-uniformity.

When different contrasts are not only combined but co-encoded, meaning the contrasts are encoded simultaneously within the same sequence, there is the potential to disentangle the signal contribution of different microstructural compartments within a voxel. For diffusion MRI, the sensitivity to both diffusivity and orientation can help dissociate MR properties of specific microstructural compartments and fiber orientations. When diffusion and relaxation are co-encoded, the relaxation times of distinct compartments and/or fiber orientations within a voxel can be measured. This is achieved by repeating the diffusion acquisition for various echo times for T_2_, or for various repetition or inversion times for T_1_. Different approaches to both the acquisition and the analysis of diffusion-relaxometry exist. For example, non-parametric signal inversion techniques sweep through a large range of experimental parameters and rely on limited assumptions to estimate the diffusivity and relaxation times of different compartments (Benjamini & Basser, 2016, 2020; de Almeida Martins et al., 2021; Kim et al., 2017) at the cost of being rather time consuming (Lampinen et al., 2020; Veraart et al., 2018). Others use compartment models to resolve the diffusivities and T_2_ relaxation values of the extra- and intra-cellular compartments (Gong et al., 2020; Lampinen et al., 2020; Veraart et al., 2018).

Biophysical and signal models can also be used to resolve the distinct properties of multiple fiber populations present within a voxel. This approach relies on assumptions about compartment properties and takes advantage of the different orientations of fiber populations to disentangle tract-specific information, such as T_1_ (De Santis et al., 2016; Leppert et al., 2021). Simultaneously estimating signal fractions, diffusion and quantitative measures for each fiber orientation within a voxel is an under-determined problem with currently available acquisitions. As an alternative to voxel-wise fitting and to simplify the problem, tract-specific properties can be estimated at the streamline or bundle level, using global optimization frameworks that make the assumption that a microstructural property (e.g., the intra-cellular signal fraction per unit length) is constant along a streamline’s length (Daducci et al., 2015). This global approach has been used to estimate tract-specific properties such as axon caliber and tract-specific intra-axonal T_2_ (Barakovic, Girard, et al., 2021; Barakovic, Tax, et al., 2021), as well as myelin water fraction (Schiavi et al., 2022).

The ability to disambiguate the microstructural features of crossing white matter tracts in the brain is especially appealing in the context of tractometry studies. In conventional tractometry, quantitative or semi-quantitative MRI (qMRI) maps are projected onto reconstructed streamlines for further analysis (Bells et al., 2011; Zhang et al., 2022). In some cases, the average profile of streamlines forming a tract is computed. This has been applied to various studies of white matter development (Yeatman et al., 2012) and pathologies such as multiple sclerosis (Dayan et al., 2016; Reich et al., 2008) and stroke (Li et al., 2022). In other applications, the scalar values from qMRI maps are averaged over the whole bundle (e.g., Correia et al., 2008; Slater et al., 2019). Such summary qMRI measures are also used to weigh the edges of structural connectomes (Boshkovski et al., 2022; Bosticardo et al., 2022; Kamagata et al., 2019; Wei et al., 2018). The tract qMRI estimates in all these studies are likely confounded by partial volume effects whereby properties from multiple fibers are averaged within a voxel. This situation is highly prevalent in white matter, where crossing and kissing fibers are present in 60-90% of voxels (Jeurissen et al., 2013) and can potentially bias the resulting measures and reduce the sensitivity to subtle differences. Incorporating tract-specific information from dual-encoded sequences could lead to more anatomically specific tract properties and more informative connectomes. However, the diffusion-relaxometry implementations described above often require time-consuming acquisitions, advanced gradient performance, and/or complex processing routines, making them less amenable for use in patient populations.

Here, we introduce an efficient dual-encoded magnetization transfer (MT) and DWI sequence to estimate the MT ratio (MTR) of individual white matter tracts using a global, whole brain optimization framework. MT is a contrast mechanism that is sensitive to the properties of bound macromolecular protons, such as bound pool fraction and exchange rate. In the brain, these bound macromolecules are largely found in cellular membranes, including the lipid-rich myelin sheath (see review Sled, 2018). Through simulations, the acquisition parameters of the dual-encoded sequence were optimized to co-encode this information in a clinically acceptable scan time of 7 minutes at 2.6 mm isotropic voxel size at 3 Tesla. The optimal protocol was executed and repeated on 10 healthy subjects and analyzed using Convex Optimization Modeling for Microstructure Informed Tractography (COMMIT, Daducci et al., 2015) to map the distinct MTR values of fiber bundles in the tractogram. The tract-specific MTR values and their scan-rescan repeatability were compared to conventional MTR tractometry.

## 2 Methods

### 2.1 Sequence design

**Fig. 1.**
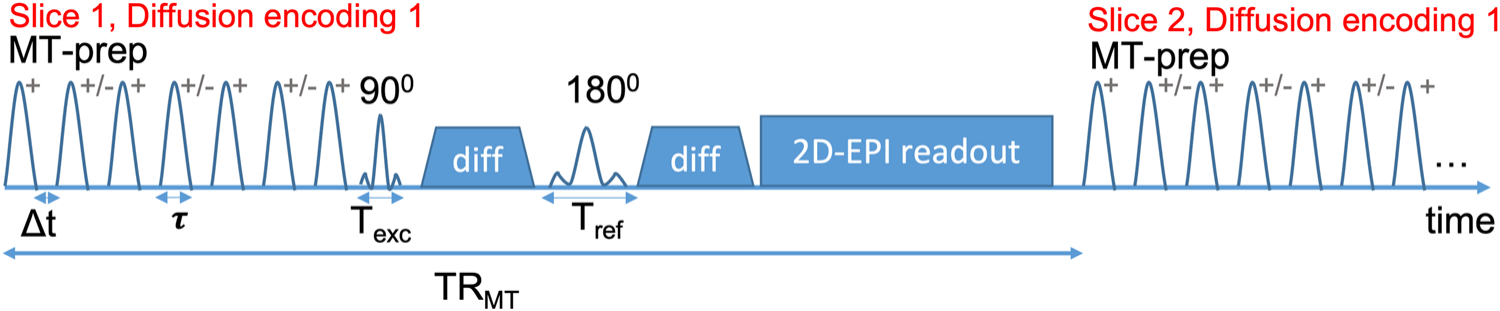
Sequence diagram of co-encoded MT-diffusion: A pulsed MT module inserted prior to the diffusion preparation of the 2D-EPI acquisition of each slice. The polarity (+/-), the duration (τ), inter-pulse time gap (Δt), number of pulses, and the flip angle of the MT pulse (FA_MT_) can be controlled as well as the duration of the excitation (T_exc_) and refocusing pulses (T_ref_). The MT preparation is repeated for each slice and each diffusion encoding. The sequence repetition time is given by TR = TR_MT_*slices.

The dual-encoded sequence comprised a spatially non-selective MT preparation module, inserted prior to the excitation of each slice in a 2D diffusion-weighted spin echo planar imaging (EPI) acquisition (Fig. 1). The MT preparation is thus repeated for each slice and each diffusion encoding. A dual-polarity pulsed MT preparation module was used to maximize contrast while maintaining an acceptable radio-frequency (RF) power deposition (Varma et al., 2018). The following parameters can be controlled by the user at the console: the offset frequency and polarity (positive or alternating), pulse duration (τ), inter-pulse time gap (Δt), number of pulses, and the flip angle of the MT pulse (FA_MT_), as well as the duration of the spin echo excitation (T_exc_) and refocusing pulses (T_ref_). Previously implemented MT-weighted spin echo (SE)-EPI sequences used either a single Gaussian off-resonance pulse combined with diffusion weighting (Gupta et al., 2005) or multiple pulses without diffusion weighting (Battiston et al., 2019).

In contrast to the typical 3D spoiled gradient echo sequences used for MT-weighted experiments, diffusion acquisitions typically use SE-EPI with fat saturation and multi-band pulses (MB), which all contribute to SAR and thus limit the energy deposition that can be used for MT contrast. The MTR is given by the ratio of the images with and without MT saturation (MTR = 1 - MT_on_ / MT_off_). This means that unwanted off-resonance contributions from the fat saturation and MB pulses will limit the available contrast since they are present in both MT_on_ and MT_off_ images. To maximize MT contrast, we chose to avoid MB acceleration at the cost of scan time. Furthermore, we investigated doing fat suppression by adjusting the ratio of the timing and amplitude of the excitation and refocusing pulses (Ivanov et al., 2010) rather than using a fat saturation pulse. These changes come at a cost of a slight increase in echo time (TE).

The number of slices and diffusion directions were chosen to provide full brain coverage and sufficient angular resolution for tractography, such that we can use a global optimization framework to estimate tract-specific MTR values (Daducci et al., 2015). We chose a relatively low b-value of 1500 s/mm^2^ to maintain sufficient signal from the extra-axonal compartment, where MT contrast will arise from the interactions at the surface of the myelin sheath. The MT-weighted signal from myelin water has mostly decayed at the relatively long TE values (~60-70 ms) required for this diffusion weighting (van Gelderen & Duyn, 2019), whereas the MT contrast from the intra- and extra-axonal spaces will remain.

### 2.2 Simulations for sequence optimization

To determine the acquisition parameters that maximize MTR efficiency, simulations of a 2-pool model, including a dipolar component, were carried out in MATLAB using recently presented optimization software (Rowley et al., 2023) following a minimal approximation approach (Portnoy & Stanisz, 2007) (model assumptions: super Lorentzian lineshape for the bound pool, longitudinal relaxation time of the bound pool T1b = 1 s, transverse relaxation of the bound pool T2b = 1 µs, dipolar order relaxation time T1d = 3 ms, transverse relaxation of the free pool T2a = 60 ms, exchange rate between the pools R = 26 s^-1^, bound pool fraction M0b = 0.1, observed relaxation time Ra_obs_ = 850 ms, Gaussian MT pulse shape).

The following parameter search space was simulated: MT offset frequency = 1-10 kHz; TR_MT_ = 90-150 ms; number of pulses = 1-15; pulse duration = 1-12 ms. The following protocol parameters were kept fixed: Δt = 0.3 ms, resolution = 2.6 mm^3^, 62 slices, TE = 58 ms, b-value = 1500 s/mm^2^, directions = 30. The FA_MT_ was set to the maximum within the SAR constraints for the whole sequence (3.2 W/kg for the head). In addition, the total scan time was constrained to a maximum of 10 minutes, which includes the two acquisitions, with and without the MT preparation module, needed to compute the MTR. MTR represents the relative decrease in signal due to the saturation pulses, which can, in part, be due to the direct saturation of water and may lead to an erroneous attribution of changes to the macromolecular pool. To maximize our sensitivity to the macromolecular pool, MTR efficiency (MTR per unit time) was computed using simulations with the 2-pool model, where MTR is the difference between two cases where M0b = 0.1, and M0b = 0, with the latter representing the free-water pool only.

### 2.3 In vivo acquisitions

All MR images were acquired on a Siemens Prisma-Fit 3 Tesla scanner using a 32-channel head coil at the McConnell Brain Imaging Centre of the Montreal Neurological Institute. The project was reviewed and approved by the Research Ethics Board of McGill University.

The simulation results were verified with two in vivo datasets: first using the optimal protocol that was found to maximize MTR efficiency (offset frequency = 3 kHz; dual irradiation; TR_MT_ = 90 ms; number of pulses = 7; pulse duration = 1 ms), and second with a higher TR_MT_ = 110 ms while keeping the total SAR constant at 97% of the allowable limit. For all subsequent in vivo acquisitions, the optimal MT preparation was used.

To verify the reduction of unwanted sources of SAR and off-resonance effects that arise from the MB excitation and refocusing pulses and fat saturation that are typically used in diffusion acquisitions, three different datasets were acquired. In the first, standard parameters were used (MB = 3, TE/TR = 55/3000 ms and fat saturation) (FS_STD+MB_); in the second, MB was removed, resulting in a longer TR (TE/TR = 55/6400 ms, fat saturation) (FS_STD_); and in the third, the fat saturation was replaced by an adjustment of the excitation and refocusing pulse length ratios that minimizes refocusing of the fat signal (FS_PL_). The pulse lengths were computed according to Eqn 8 in Ivanov et al. (2010) for a field strength of 3 Tesla and a slice thickness of 2.6 mm (TE/TR = 58/5900 ms, T_exc_ = 3.328 ms, T_ref_ = 9.472 ms). In all cases, the MT preparation was kept the same (optimal based on simulations), the TE and TR were set to the minimum, and the FA_MT_ was increased until the total SAR reached 97% of the allowable limit. All other parameters were kept constant (63 slices, GRAPPA = 2, resolution = 2.6 mm isotropic, PF = 6/8, BW = 1500 Hz/px, b-value = 1500 s/mm^2^). To assess the difference in contrast-to-noise ratio (CNR) and signal-to-noise ratio (SNR), 10 b = 0 images were acquired for each condition and the MTR was compared in white and gray matter regions of interest (ROI). Both the average and standard deviation were first done across the 10 MTR b = 0 images, then averaged across the regions of interest. An additional source of unwanted MT effects stems from the multi-slice acquisition, whereby neighboring slices experience an off-resonance contribution while the current slice is being excited. To quantify this contribution, we acquired a single-slice b = 0 MT_off_ dataset with all other parameters matched to the multi-slice acquisition.

The optimal parameter combination was used to scan 10 healthy subjects (4 women, aged 30 ± 10 years) over 2 separate sessions to assess scan-rescan repeatability. The acquisitions included the optimal MT-diffusion (MT_on_ and MT_off_), a reverse phase-encoded b = 0 scan for distortion correction, and a T1-weighted MPRAGE for registration and brain tissue segmentation (1 mm isotropic, GRAPPA = 2, TE/TI/TR = 2.98/900/2300 ms, FA = 8°). All sequences used the adaptive combine coil combination algorithm.

### 2.4 Image pre-processing

Diffusion-weighted images (DWIs) (both with and without MT saturation) were combined into a single volume and denoised (Veraart et al., 2016) and pre-processed to account for subject movement, susceptibility, and eddy current induced distortions with a combination of MRtrix3 (Tournier et al., 2019) and FSL (Andersson et al., 2003). Image non-uniformity correction was performed with ANTs (Tustison et al., 2010), using the bias field estimated from the MT_off_ data applied to both the MT_off_ and MT_on_ data; the same correction was used for both acquisitions. Non-uniformity correction is necessary for COMMIT because this global optimization approach relies on the signal being uniform along streamlines. Subsequently, all DWIs were up-sampled to 1 mm isotropic voxels, registered to the T1-weighted MPRAGE image, and aligned to the Desikan-Killiany segmentation atlas using FreeSurfer v7 (Desikan et al., 2006). The brainstem was further segmented into substructures (Iglesias et al., 2015). The computed transformations were inverted and applied to the T1-weighted image and the atlas such that all further analysis was carried out in the subject’s native diffusion space. The response functions for single-fiber WM as well as GM and CSF were estimated from the data using an unsupervised method (Dhollander et al., 2019). Single-Shell 3-Tissue CSD (SS3T-CSD) was performed to obtain WM-like fiber orientation distributions (FODs) as well as GM-like and CSF-like compartments in all voxels (Dhollander & Connelly, 2016) using MRtrix3Tissue (https://3Tissue.github.io), a fork of MRtrix3 (Tournier et al., 2019). Anatomically constrained probabilistic tractography was performed on the MT_off_ data only using the iFOD2 algorithm (Tournier et al., 2010) with 3 million streamlines, as implemented in MRtrix3. Streamlines not connecting nodes of the atlas were discarded.

### 2.5 Tract-specific MTR analysis pipeline

COMMIT was used to estimate tract-specific MTR values as follows. COMMIT estimates a chosen parameter describing a microstructural property of a streamline from tractography with the assumption that this parameter is constant along the streamline’s trajectory. This parameter is called the streamline weight, *x*. In the current implementation, the streamline weight is the signal per unit length of the part of the fiber bundle represented by the streamline. Ignoring sources of signal variation such as relaxation and macromolecular content, this can be interpreted as a volume per unit length, that is, the cross-sectional area (Smith et al., 2022). Here, the compartments of the fiber bundle attributed to the streamline consist of the combined intra- and extra-axonal spaces of the fiber. The diffusion response function of this combined space can be represented by an anisotropic tensor or “zeppelin.” The other space in the voxel is represented by a “ball” modeling free water (Panagiotaki et al., 2012) and can vary from voxel to voxel. It is therefore assumed that the signal contribution from the intra-axonal space and its immediate surrounding extra-axonal space is constant along each streamline. Furthermore, all streamlines throughout the brain are assumed to have the same diffusivity parameters (diffusivity parallel to the streamline direction D_||_ = 1.7E-3 mm^2^/s; diffusivity perpendicular to the streamline direction D_┴_ = 0.6E-3 mm^2^/s) and the ball compartment (isotropic diffusivity = 3E-3 mm^2^/s but will capture either free water or isotropic cellularity such as gray matter). In this context, the ball compartment is meant to be discarded and omitted from further analysis. COMMIT was applied separately to the MT_on_ and MT_off_ datasets, using the tractogram computed on the MT_off_ dataset. In both cases, the signal was normalized to the b = 0 s/mm^2^ of the MT_off_ dataset. The drop in signal due to MT-weighting will lead to a proportional change in streamline weights between the two fits. The following voxel-wise equations describe the fitting process of the MT_on_ and MT_off_ datasets:

$$\frac{S\left(q,MT_{off}\right)}{S\left(b=0,MT_{off}\right)}=\sum_{i}x_{i}^{zeppelin,\;MT_{off}}R_{i}^{zeppelin}(q)+x^{ball,MT_{off}}$$ Eq.1

$$\frac{S\left(q,MT_{on}\right)}{S\left(b=0,MT_{off}\right)}=\sum_{i}x_{i}^{zeppelin,\;MT_{on}}R_{i}^{zeppelin}(q)+x^{ball,\;MT_{on}}$$ Eq. 2

where S(**q**) is the signal at each q-space location, R^zeppelin^ represents the response function of the zeppelin compartment, rotated to align to the fiber orientation, and is scaled by the length of the streamline intersecting the voxel. Finally, the x^zeppelin^ represents the contribution of each streamline (*i*) and x^ball^ the contribution of free water in the voxel. Streamlines whose weight was greater than zero in both MT_on_ and MT_off_ were grouped into bundles according to the pair of gray matter regions they connect. The tract-specific MT_on_ and MT_off_ were then calculated as the sum of all streamline volumes in the bundle. The volumetric contribution of streamline *j* to the bundle is given by the product of its weight *x_j_* times its length *L_j_*. Finally, the tract-specific MTR is computed by combining the individual connectomes with the standard equation (Schiavi, Ocampo-Pineda, et al., 2020):

MTRbundle=1−MTonbundleMToffbundle=1−∑jNLj⋅xjzeppelin,MTon∑jNLj⋅xjzeppelin,MToff Eq. 3

A selection of large white matter tracts that connect nodes of the Desikan-Killiany atlas were extracted from the final connectome. The selected tracts are listed in Table 1 along with the nodes they connect.

**Table 1.** Selected bundle names, description, and nodes of the Desikan-Killiany parcellation that they connect.

Name	Description	Name of brain region and (node number)
fWM	Pathways connecting the bilateral frontal white matter, passing through the genu of the corpus callosum	Left (27) to right (76) superior frontal cortex
SLF	Superior longitudinal fasciculus	Left (27) superior frontal to left (28) superior parietal cortexRight (76) superior frontal to right (77) superior parietal cortex
Pons	Pontine fibers	Left (35) and right (84) cerebellar cortex
CST	Pathways of the corticospinal tract that connect the brainstem and motor cortex	Brainstem to left (23) and right (72) precentral gyrus
PrCG-Thal	Projection pathway between the precentral gyrus and thalamus	Left precentral gyrus (23) to left thalamus (36)Right precentral gyrus (72) to right thalamus (43)
Splen	Pathways connecting the bilateral occipital white matter, passing through the splenium of the corpus callosum	Left lateral occipital cortex (10) to right lateral occipital cortex (59)

**Fig. 2.**
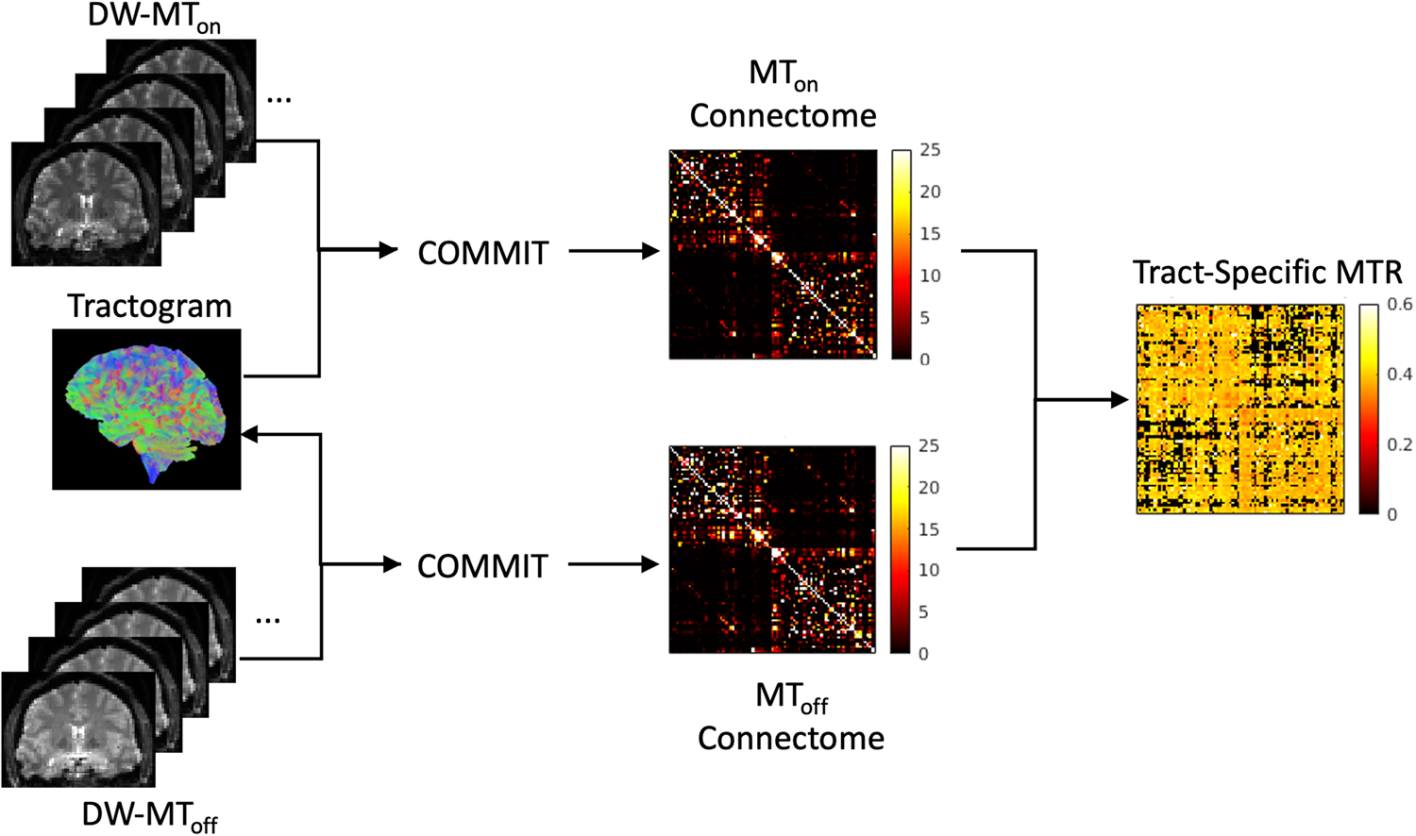
Processing pipeline: The MT_off_ data are used to generate the tractogram, which is then used with COMMIT to produce the MIT_on_ and MT_off_ connectomes. These connectomes are then combined to get tract-specific MTR values using Eqn 3.

The pipeline is illustrated in Figure 2.

As previously done in Schiavi et al. (2022), the proposed method was compared to what we will refer to henceforth as conventional tractometry, whereby a separate MTR map was sampled along each streamline, taking the median along its length, and averaged across streamlines within a bundle. The MTR maps used here were calculated as the average across all directions of the diffusion-weighted datasets, omitting the b = 0 image (MTR_dw_). For both methods, subject-wise bundle MTR values were compared with a t-test, to quantify whether there were consistent differences between bundles. The full pipeline is available on GitHub (https://github.com/TardifLab/mt-diff) and sample data are available through Dataverse (https://doi.org/10.5683/SP3/LNFHGO) or upon request through a formal data sharing agreement and approval from the local ethics committees.

## 3 Results

### 3.1 Sequence optimization

**Fig. 3.**
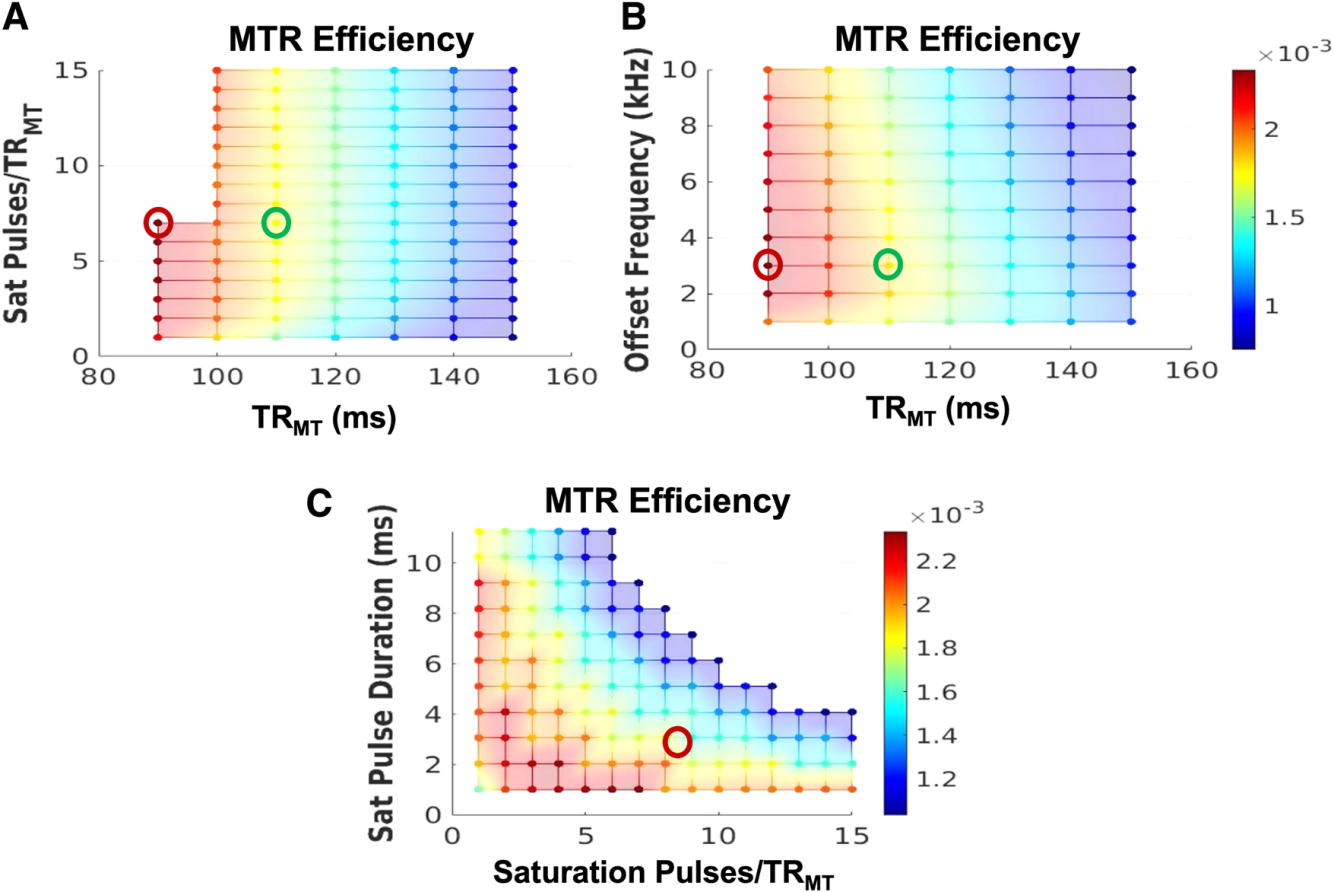
Simulated results of MTR efficiency: (A) Number of saturation pulses vs TR_MT_ (B) Offset frequency vs TR_MT_ and (C) Saturation pulse durations vs number of saturation pulses. Red circle highlights the optimal protocol and the green, sub-optimal.

Based on the simulation results (Fig. 3), our optimal protocol was: offset frequency = 3 kHz; dual irradiation; TR_MT_ = 90 ms (minimum); number of MT pulses = 7; pulse duration = 1 ms. These results point to TR_MT_ being the parameter with the most significant impact on MTR efficiency. This is seen in Figure 3A and B, where for a fixed TR_MT_, several different combinations of offset frequencies and number of pulses lead to a similar efficiency. This is why we chose to validate the simulation results by acquiring two datasets, first using the optimal protocol with TR_MT_ = 90 ms (red circle in Fig. 3), and second at a higher TR_MT_ = 110 ms (green circle) while keeping the total SAR constant at 97% of the allowable limit. This resulted in an FA_MT_ of 596° and 656° respectively.

**Fig. 4.**
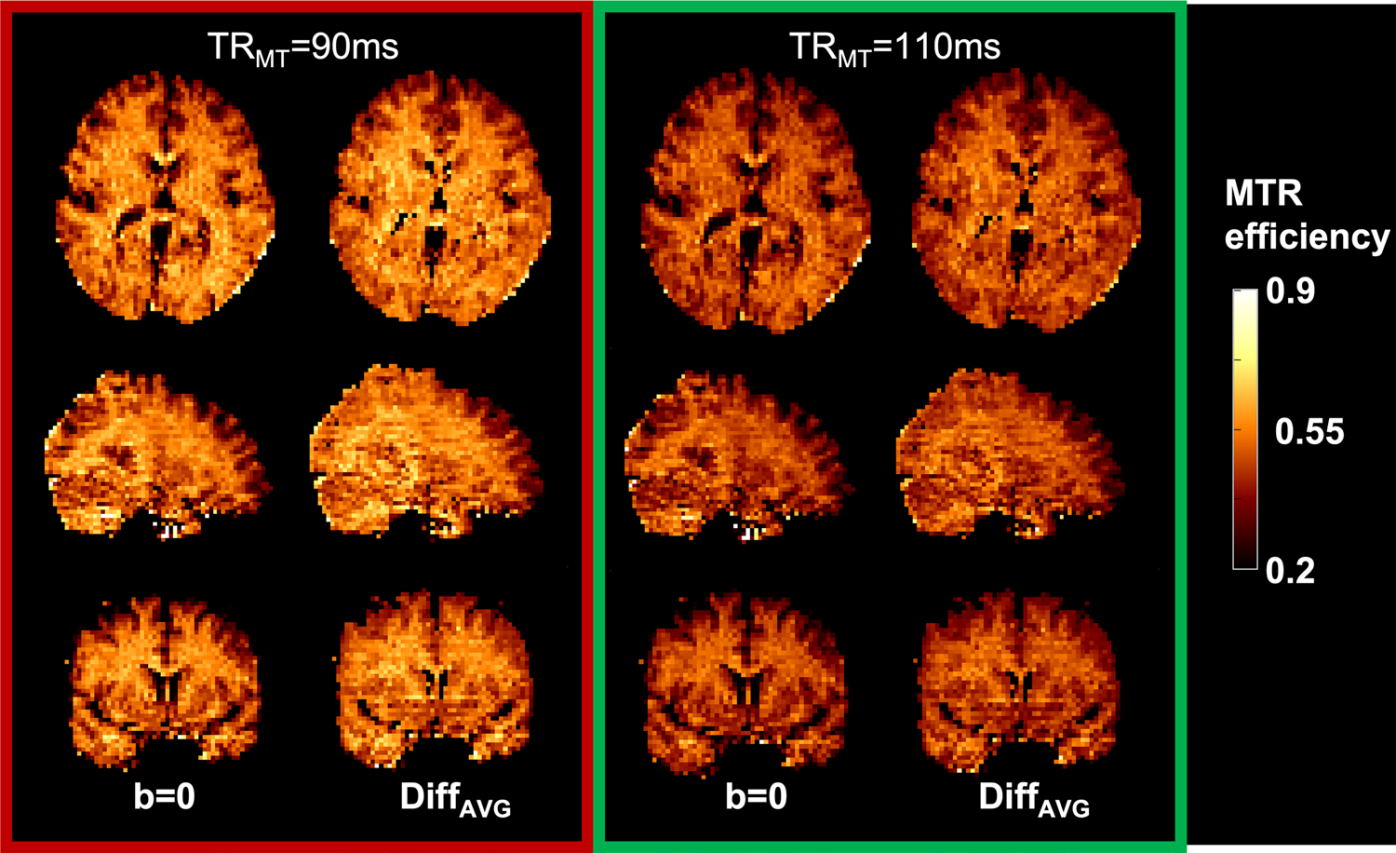
MTR efficiency (MTR/time) of optimal (TR_MT_ = 90 ms, red outline) and sub-optimal (TR_MT_ = 110 ms, green outline) protocols of the b = 0 and of the average over 30 diffusion directions (DiffAVG).

Figure 4 illustrates the MTR efficiency (MTR/time) calculated using the b = 0 images with and without MT-weighting, as well as for the average of the 30 diffusion orientations (Diff_AVG_). This agrees with the simulation results, where minimizing the TR_MT_ increases the MTR efficiency.

**Fig. 5.**
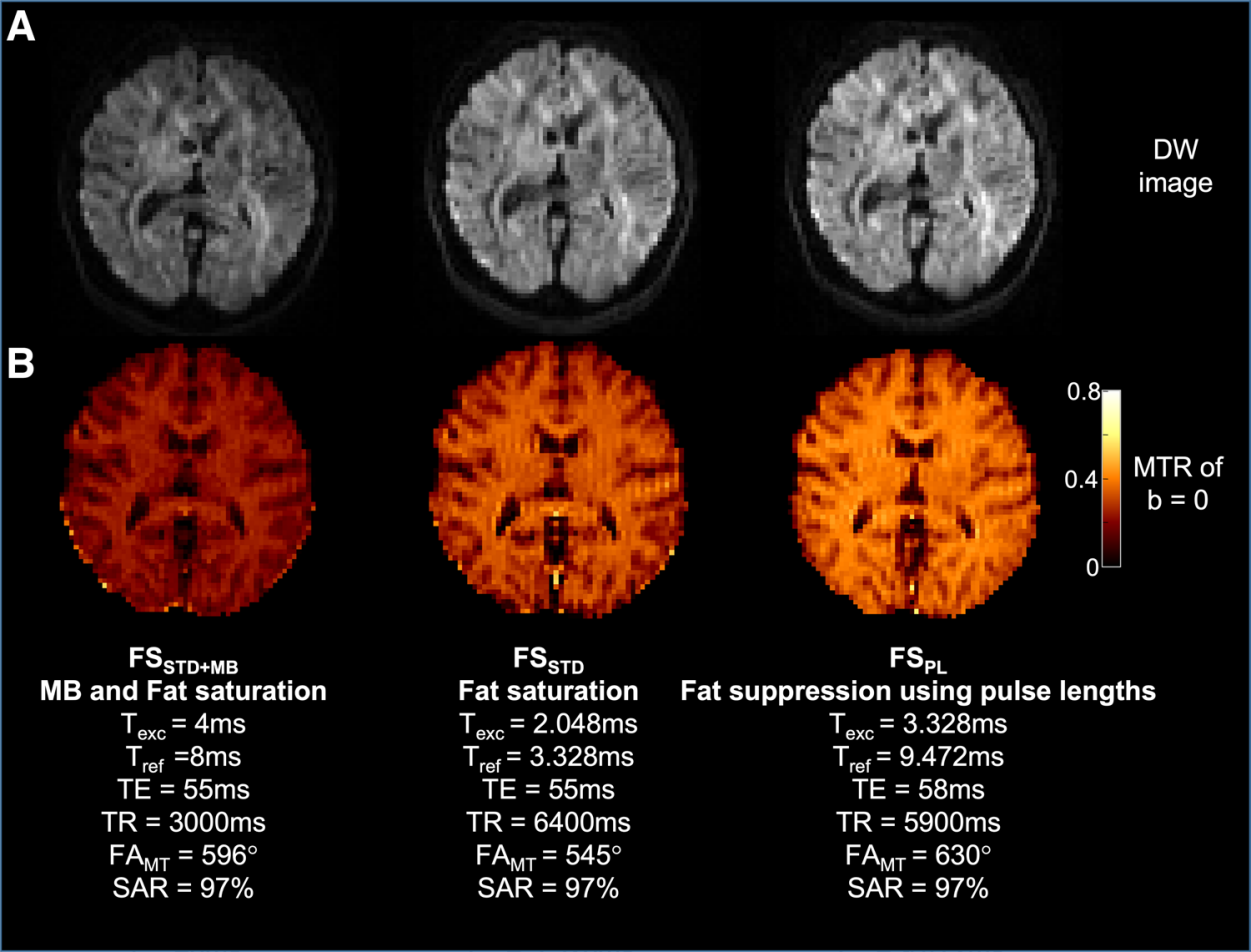
Reducing sources of unwanted SAR and MT by avoiding MB and replacing standard fat saturation by using a ratio of pulse lengths for fat suppression. (A) diffusion-weighted image (B) the MTR of the b = 0 images.

As shown in an example diffusion-weighted image (Fig. 5A), the scalp fat signal was successfully suppressed with a T_exc_ = 3.328 ms and T_ref_ = 9.472 ms for a slice thickness of 2.6 mm, at the cost of a slight increase in TE (+3 ms) compared to the standard protocol using fat saturation. The TR is effectively reduced with the fat shifting technique, since the 5.1 ms fat saturation pulse prior to each slice is removed (6400 ms vs 5900 ms). Figure 5B illustrates the gain in MTR (68%) that can be achieved by removing MB and the standard fat saturation, whereby the FA_MT_ can be increased while remaining within 97% of the allowable SAR limit. To accommodate differences in participant heights and weights, the FA_MT_ was lowered to 580° (from 596°) for the subsequent in vivo acquisitions such that for all participants, MT was maximized without the SAR exceeding the allowable limit (97 ± 3%).

The MTR CNR between gray and white matter is highest in the case of using fat saturation and different pulse lengths (FS_STD_ = 6.0, FS_PL_ = 5.6), compared to using fat saturation with multi-band (FS_STD+MB_ = 4.6). The MTR SNR is highest for FS_PL_ (SNR = 27) compared to using FS_STD_ and FS_STD+MB_ (SNR = 23 and 14). This implies that although FS_PL_ did not provide additional gray-white MTR contrast compared to FS_STD_, this method did provide an increase in SNR compared to the other methods.

Finally, the unwanted but unavoidable off-resonance contribution from the multi-slice compared to a single-slice acquisition was on average 25% (Fig. S1). Despite this, as shown in Figure 5, we can still achieve an MTR of ~40% in the white matter using a multi-slice sequence.

### 3.2 In vivo comparison of tract-specific and tractometry MTR

**Fig. 6.**
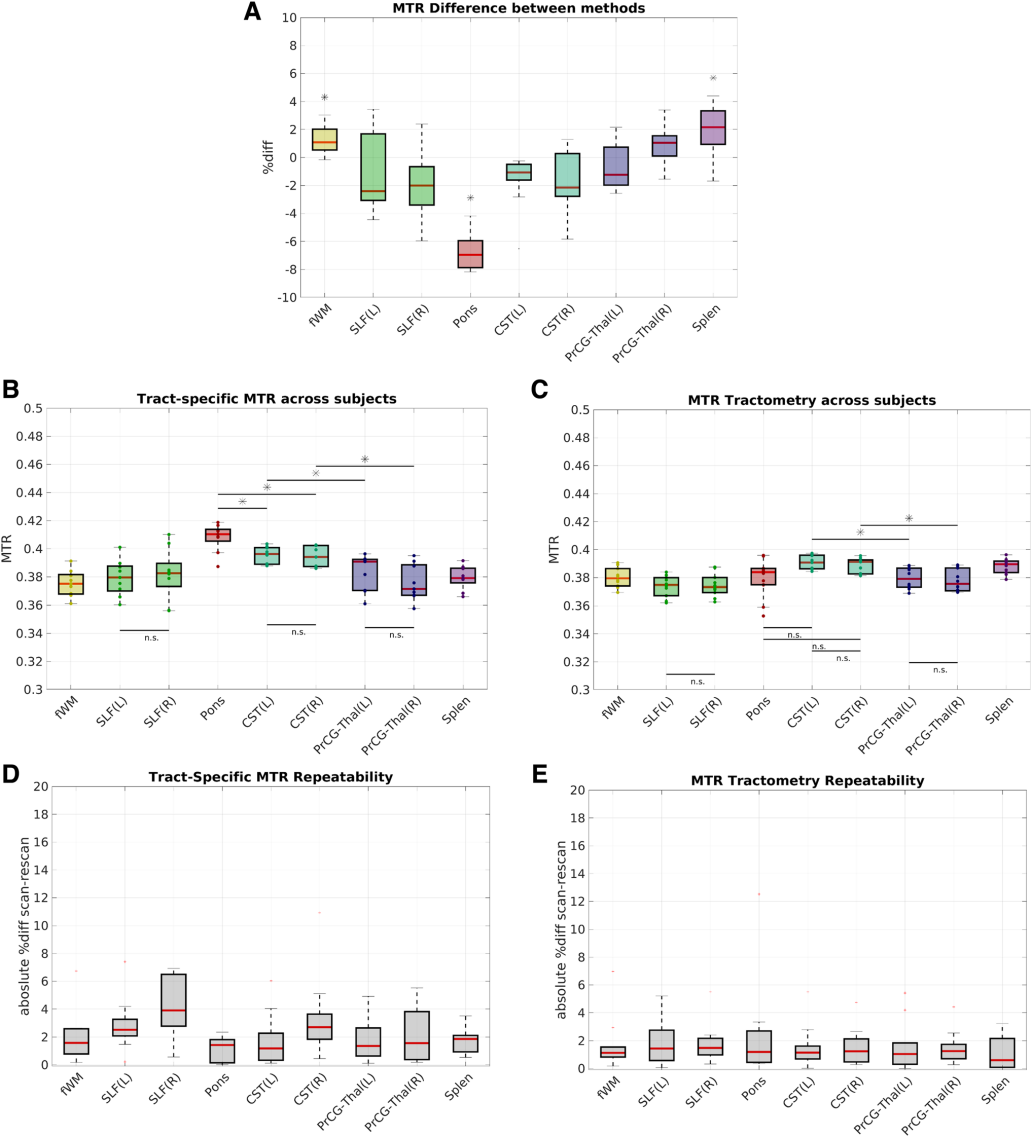
(A) Percent difference between tract-specific and tractometry MTR for selected bundles (B) Average tract-specific MTR (C) Average tractometry MTR and (D,E) corresponding scan-rescan repeatability across 10 subjects (* denotes significance at p < 0.01; n.s. not significant).

The results for tract-specific and tractometry MTR values across all 10 subjects are shown in Figure 6 for the selected white matter tracts listed in Table 1. As shown in (A), there are significant within-subject differences between the methods for the fWM, Pons and Splen (shown with an *, p < 0.01). In (B) and (C), the group-wise MTR values are shown for both methods, as well as the corresponding scan-rescan repeatability in (D) and (E). The mean scan-rescan percent difference of tract-specific MTR in the selected tracts is slightly higher than that for tractometry (~3% vs ~1%) and as expected, the bilateral differences (left (L) and right (R)) are not significant (n.s.) for both methods. However, the tract-specific MTR exhibits a higher dynamic range and a significant difference between the pons and CST which is not present for the standard tractometry method. For both methods, there is a significant difference between the CST and PrCG-Thal connections.

**Fig. 7.**
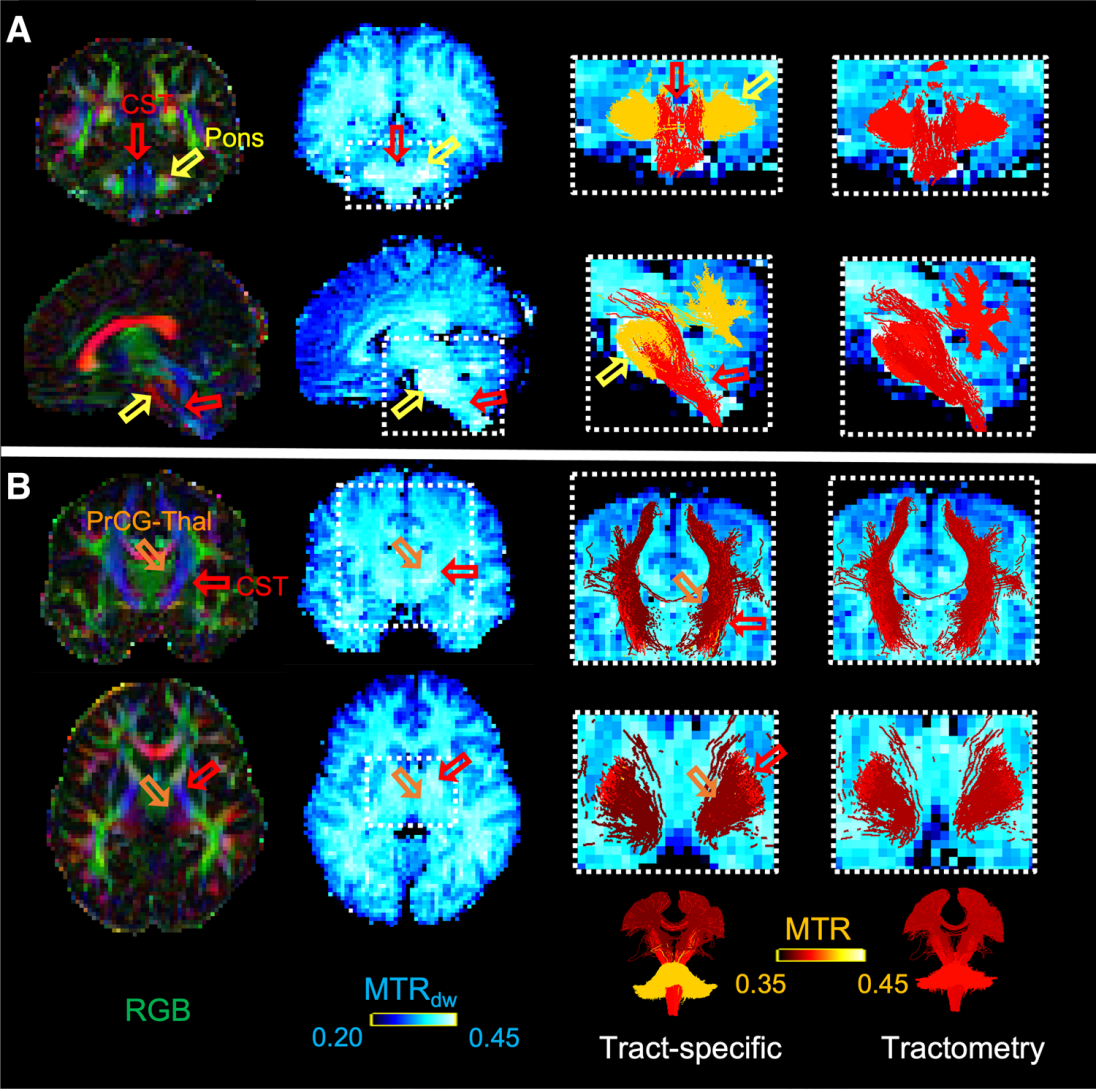
Example of tract-specific and tractometry bundle MTR results for one subject. Open arrows highlight the regions where a single fiber population is dominating the voxel (e.g., in (A): the pons (yellow) and CST (red) and (B): the PrCG-Thal connection (orange)), which gives an indication of the non-partial-volumed MTR_dw_ value along each tract (blue color scale). The overlayed MTR results (hot color scale) for the tract-specific method show better agreement with the underlying scalar map and higher contrast between tracts compared to conventional tractometry.

**Fig. 8.**
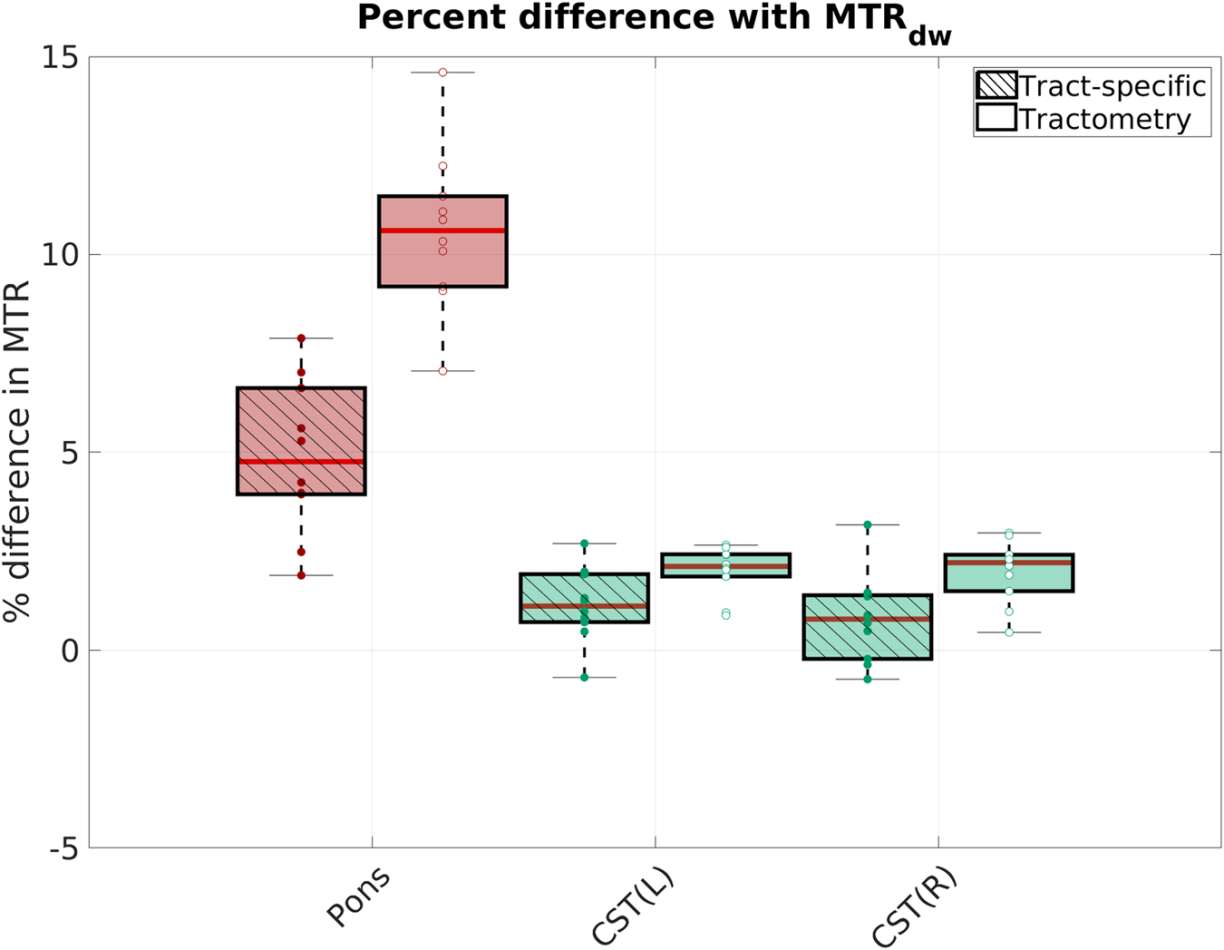
Percent difference between single-fiber voxel MTRdw values and tract-specific MTR and tractometry MTR values, respectively for the pons and bilateral cortico-spinal tracts.

An example of these intersecting tracts is shown in Figure 7, where the difference in tract-to-tract variability between the two methods is evidenced. The MTR results are overlayed on the MTR_dw_ map for a single subject, echoing the trend in tract-specific MTR values of Figure 6B (Fig. 7A: MTR_pons_>MTR_CST_; Fig. 7B: MTR_CST_>MTR_PrCG-Thal_). The zoomed-in boxes in Figure 7 highlight regions where a single tract population is dominating the voxel, such that the value in the underlying MTR_dw_ map gives a good indication of the expected MTR along its length. For example, the yellow open arrows in panel A point to voxels where the pons is the dominating fiber population and the red arrows point to voxels where the CST is the dominant population, such that the corresponding scalar MTR_dw_ is indicative of the non-partial-volumed MTR of the tract, where there are higher values in the pons than in the CST (blue color scale). This was further verified for each subject, by selecting the single fiber voxels using an FA threshold of 0.6 and the corresponding MTR_dw_ values were sampled along the streamlines of the CST and Pons. A weighted average was then computed using the tract density. Each subject’s tract-specific and tractometry values were compared to the corresponding single-fiber MTR_dw_ values. The results in Figure 8 show that the tract-specific MTR is in better agreement (mean % difference: Pons = 4.9%; CST(L) = 1.1%; CST(R) = 0.81%) compared to the tractometry results (mean % difference: Pons = 10.6%; CST(L) = 2.0%; CST(R) = 2.0%), which show less difference in MTR values across bundles. This is likely due to the reduction in partial volume effects at the intersection of the tracts, provided by the tract-specific method.

We assume a single zeppelin response across all streamlines while trying to estimate differences in bundle myelin content; however, the diffusion response function may in some cases change when myelination changes. To verify that the MTR differences we reported are robust to errors in the assumption of the shape of the zeppelin, the COMMIT analysis was repeated in a single subject, varying the shape of the zeppelin by using a range of fractional anisotropy values (FA = 0.4-0.8) in healthy white matter. This range should both over- and under-estimate the anisotropy of each bundle. As shown in Figure S2, there is very little change in the overall trend and differences between bundles despite the large range of FA values. This suggests that even if there is significant error in the diffusion response, the MTR differences between bundles are still being captured.

In terms of the reliability of the tractography and two-compartment model given the relatively succinct acquisition (single-shell, 30-directions, relatively low b-value of 1500 s/mm^2^), we investigated the quality of the fit and FODs. In the COMMIT framework, the signal fraction that is not assigned to the “zeppelin” nor the “ball” compartment is reported through the RMSE (root-mean-squared error) of the fit. As shown in Figure S3, the “ball” or isotropic (ISO) compartment is essentially zero throughout the white matter, while the RMSE is relatively homogeneous for both MT_off_ and MT_on_. At the boundary of both the ventricles and gray matter, the “ball” compartment is non-zero since all sources of isotropic diffusion are captured in this parameter (gray matter and CSF cannot be distinguished). The difference between the RMSE of MT_off_ and MT_on_ is homogeneous and small compared to the RMSE, indicating that although using a fixed zeppelin is not ideal (and may not reflect reality), the impact on the resulting tract-specific MTR estimates is small.

Although using a low b-value with a limited number of directions is not the optimal acquisition for resolving crossings, many previous publications using lower b-value acquisitions have been able to reliably detect crossings (Behrens et al., 2007; Descoteaux et al., 2009; Parker & Alexander, 2003). This would particularly be the case in the major fiber pathways that have been presented in this manuscript. In addition, recent work has shown that multi-tissue FODs can be estimated from single-shell data (Dhollander & Connelly, 2016). The relatively low b-value was chosen to maintain signal and MTR contrast from the extra-cellular compartment, and the limited number of directions was chosen to save on scan time. Without this time constraint, it would be possible to use the streamlines reconstructed from a separate, more extensive acquisition (more shells and directions), but the differences in distortion which follows from longer TE acquisitions would have to be carefully addressed. To verify that the FODs are reliable, we have acquired a high angular multi-shell (b-value [s/mm^2^] (directions): 0(6), 300(10), 1000(30), 2000(64)) in the same session as the dual-encoded acquisition (b-value [s/mm^2^] (directions): 0(1), 1500(30)). Figure S4 shows an example of the orientation distribution functions in a zoomed-in section of the CST and pontine tract crossing, where, similarly to the multi-shell acquisition, the single-shell can distinguish the two populations. Although the relative sizes of the lobes are slightly different, the use of probabilistic tractography followed by COMMIT filtering to help remove false positives should lead to similar tractograms, especially in large tracts.

## 4 Discussion

The goal of this work was to design and optimize an efficient dual-encoded sequence, which can be combined with a global optimization microstructure informed tractography framework to estimate tract-specific MTR values. One of the primary motivations is to be able to provide tract-specific myelin indices, which should, in turn, lead to more anatomically and microstructurally specific myelin-weighted structural connectomes. This is achieved by effectively reducing the contamination caused by partial volume averaging along tracts, such that connectome edge weights reflect the MTR of the associated bundle exclusively.

In terms of the sequence design, our simulation results agree with previous work from Varma et al. (2018) where the use of dual irradiation and rapidly switching between polarities, in this case short 1 ms pulses with alternating polarity, help maximize MTR (Lee et al., 2011; Varma et al., 2017). In addition, we showed that by not using MB pulses and fat saturation, more power can be used for MT contrast, at the cost of an increase in scanning time. Nevertheless, a protocol optimized for MTR efficiency with both MT_on_ and MT_off_ can be acquired in under 7 minutes.

In line with previous literature (Garcia et al., 2011; Mehta et al., 1995), the range of MTR values in major white matter fiber tracts in healthy young adults is relatively small. This and the low number of subjects (10) might explain the limited number of significant differences between tracts at the subject level for both the tract-specific and tractometry methods. However, in the case of the tract-specific values, the dynamic range is larger, and the group trends are corroborated by the MTR_dw_ map, particularly when referring to regions where a particular tract is the dominating population in the voxel. Conversely, the tractometry maps are relatively flat, likely due to the extensive partial volume effect occurring over the length of the tracts. The increase in dynamic range has also been seen in previous work using both voxel-wise and global approaches compared to more standard tractometry (De Santis et al., 2016; Leppert et al., 2021; Schiavi et al., 2022).

We have not found previous histology literature with tract-specific MTR values in human healthy white matter. ROI-based measures in healthy animals (e.g., Duhamel et al., 2019; Guglielmetti et al., 2020; Hakkarainen et al., 2016) and cortical myelination patterns in human subjects (Nieuwenhuys & Broere, 2017) have been reported, but are not ideal to validate our tract-specific MTR results. Therefore, we have focused on tracts where single-fiber voxels can be isolated to validate the tract-specific MTR values that were estimated, based on the underlying contrast that was acquired (diffusion-weighted MTR). These results echo our previous work with tract-specific T1 relaxometry (Leppert et al., 2021), where the pontine tracts exhibited a lower T1 than the CST. We hypothesize that by reducing the partial volume effects inherent to MRI, a more anatomically specific MTR can be extracted. The scan-rescan repeatability is lower in the tract-specific approach compared to the tractometry. This is expected because with COMMIT, we are using a signal model to fit streamlines with the goal of estimating tract-specific properties, whereas with tractometry, no fitting is involved, and the resulting tract information is averaged over all the voxels in the qMRI map that underlie the bundle. The averaging across diffusion-weighted images (MTR_dw_) and over tracts effectively acts as a blurring kernel, reducing noise and making measurements more repeatable. The gain in specificity with the tract-based method, which uses each diffusion direction as a separate measurement, unfortunately also leads to an increase in noise sensitivity and/or fitting error. Sources of error that particularly affect the global optimization technique are false positives and false negatives in tractography and the sensitivity to B1^+^ non-uniformity. Future work will focus on exploring model-based corrections for B1^+^ non-uniformity (Rowley et al., 2021) and the reduction of T_1_ bias in the MTR maps (Helms et al., 2008).

Previous work on estimating tract-specific measures includes co-encoded voxel-wise fitting of myelin-sensitive T_1_ (De Santis et al., 2016), co-encoded global tract-based analysis of axon caliber (Barakovic, Girard, et al., 2021) and intra-axonal T_2_ (Barakovic, Tax, et al., 2021), as well as global tract-based analysis to estimate bundle-specific myelin content using separate acquisitions (MTsat and myelin water fraction) for myelin contrast (Schiavi et al., 2022). The current implementation aims to draw advantages from these previous methods while attempting to simplify both the acquisition and analysis by using fewer measurements and a simple ratio of contrasts with global tractograms, while still providing co-encoded information. One advantage of the global optimization technique over voxel-wise approaches is its ability to dissociate bundles that are parallel at the voxel level for some extent of their trajectory and then diverge, becoming geometrically distinct globally. The co-encoding approach presented here is expected to provide additional potential for bundle dissociation, particularly when there are large expanses of voxels with multiple bundles, which is the case for most of the voxels in the brain (Jeurissen et al., 2013). If partial volume effects were not an issue, tractometry would theoretically have given the same results. Although standard diffusion and in particular GRE-based MTR maps can be individually acquired at higher resolution and would suffer from less PVE, co-encoding MT and diffusion allows us to disentangle MTR values at the streamline level, such that the information per voxel is effectively increased compared to standard single-encoded sequences. In other words, it helps minimize PVEs that occur along tracts caused by multiple fibers co-occurring in voxels along their length and potentially assigns more specific weights to streamlines and connectomes. Even if it were possible to significantly increase the resolution of the diffusion images, the prevalence of complex fiber geometry seems to increase, such that this is the limiting factor rather than limits of the imaging hardware (Schilling et al., 2017). Finally, in terms of co-registration, the different distortions in EPI-based readouts compared to single k-space line GRE readouts make it difficult to achieve perfect co-registration and co-localization, which is an added benefit of co-encoding. COMMIT can dissociate parallel tracts because of its global cost function, which attempts to fit the signal in all voxels simultaneously, combined with the assumption that the microstructure of a streamline is constant along its length.

The choice of the zeppelin & ball model was made primarily because the MT effect is expected to be significant in the extra-axonal space, and we want to capture all of this. Unlike other implementations of COMMIT (Daducci et al., 2015) that keep the intra-axonal compartment signal constant along all streamlines and the extra-axonal compartment varies per fixel (fiber element in a voxel), we chose to keep the intra- plus proximal extra-axonal compartments constant. This implies that changes in fiber density within a single streamline are minimal or have a minimal effect on the diffusion signal.

Although the MTR contrast reported here will correlate with myelin density to some extent, it will be modulated by the surface-to-volume ratio, sheath thickness, and exchange between compartments, and thus be sensitive to fiber size, the presence of other bound proton populations, orientation, and packing geometry as well. In fact, the source of the MTR contrast is different than traditional MT-GRE experiments, whose short TE is geared towards weighting interactions at both the inner and outer surface of the myelin as well as within the sheath in order to estimate myelin content. With a longer TE and diffusion weighting, the MT-weighted signal is reduced overall, but to a different extent for each compartment. The echo time will modulate the contribution of each compartment based on their respective T_2_, such that the myelin water (T_2_ = 10-40 ms) (MacKay et al., 1994) signals contribute the least and the extra-axonal (T_2_ = 30-50 ms) and intra-axonal (T_2_ = 80-120 ms) (Veraart et al., 2018) signals contribute the most. Diffusion weighting will have a greater attenuation on the extra-axonal water than the intra-axonal compartment. However, the relative contribution to the MT effect of each compartment and the extent to which the contrast is myelin-weighted, as well as the role of water exchange are not fully understood. Very recent work in fixed tissue (Li et al., 2023) suggests that the intra-axonal compartment is more affected by MT than the extra-cellular compartment. The MT-diffusion experiment is inherently less affected by unwanted direct saturation effects, due to the attenuation of free water by diffusion and the fact that it is included in a separate compartment (ball). Therefore, through the modulation of TE and b-value, the contributions of the different compartments to the tract-specific MTR measurements can be explored. Centric k-space encoding techniques and powerful gradients can be used to shorten the TE and increase the achievable b-value to gain a more detailed understanding of the origin of the MT- and diffusion-weighted signal.

Ultimately, many of the methods discussed above provide complementary tract-specific measures of microstructure that could be combined to understand the role of tract microstructure on brain connectivity and function. It may be particularly useful in detecting subtle differences between tracts that may be altered during neurodevelopment, aging (Slater et al., 2019) or through disease (e.g., schizophrenia (Wei et al., 2018)). Pathologies including focal demyelination are not the intended application for this technique. Similar to any technique for generating connectomes that averages qMRI estimates within tracts (e.g., tractometry), the extent and nature of the focal abnormalities will determine whether they are detectable or not. We have added additional analysis investigating the tensor shape and its impact on MTR estimates (Fig. S2). These results show that the tract-specific MTR values are relatively unaffected by tensor shape, meaning that although the standard zeppelin shape might not fully represent the data in focal lesions, the MTR difference would still be measurable, provided it affects a significant extent of the tract. Future work will investigate explicitly modeling tissue damage to increase the robustness to focal abnormalities (Bosticardo et al., 2023). Future work will also include comparing the performance of the new methods across different modalities and applications.

## 5 Conclusion

This work presents a novel dual-encoded MT and diffusion sequence, for which parameters have been optimized for MTR efficiency. The resulting 2.6 mm whole brain protocol can be acquired in under 7 minutes and is an important step towards providing tract-specific myelin indices that minimize biases due to partial volume effects with neighboring tracts. Future work will investigate how this may provide more statistical power and insight when the microstructure of specific tracts is altered, for example, through disease, aging, function, or treatment. Finally, the potential to provide more anatomically specific connectomes could have a significant impact on brain network analysis.

## Supplementary Material

Supplementary Material

## Data Availability

The full pipeline is available on GitHub (https://github.com/TardifLab/mt-diff), and sample data are available through Dataverse (https://doi.org/10.5683/SP3/LNFHGO) or upon request through a formal data sharing agreement and approval from the local ethics committees.
