# Supplementary material for: Dual-encoded magnetization transfer and diffusion imaging and its application to tract-specific microstructure mapping
Source: Imaging Neurosci (Camb). 2023 Sep 26;1:imag-1-00019. doi: 10.1162/imag_a_00019 (PMC12007536; doi:10.1162/imag_a_00019)
Supplement: Supplementary Material [file imag_a_00019-supp.pdf]

Supplementary Material

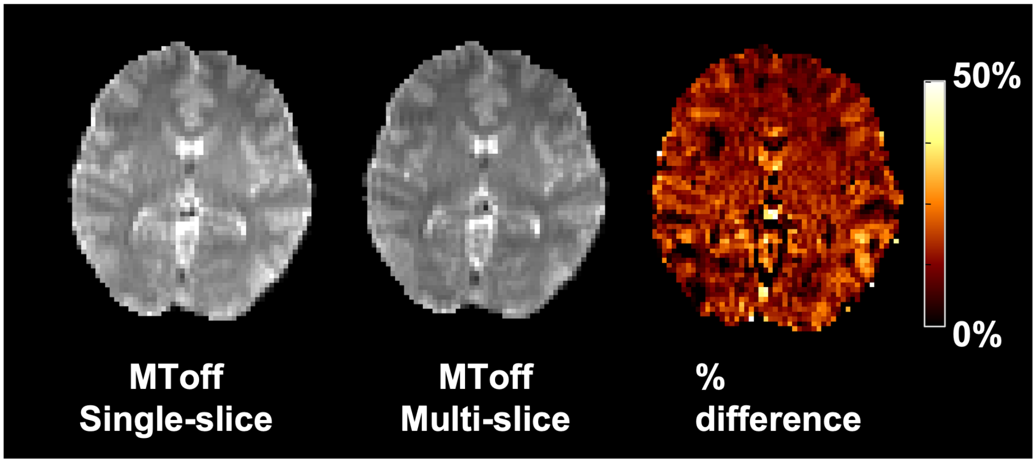

Figure S1: Off-resonance contribution of multi-slice acquisition: comparison of the b=0 MT_{off} between single-slice and multi-slice acquisitions

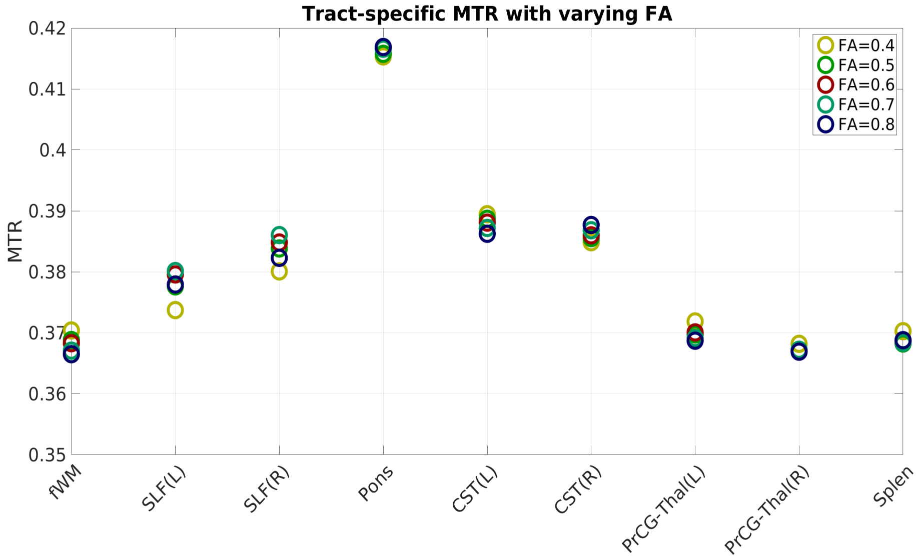

Figure S2: Tract-specific MTR values for a single subject using COMMIT with a range of different zeppelin shapes (FA=0.4-0.8)

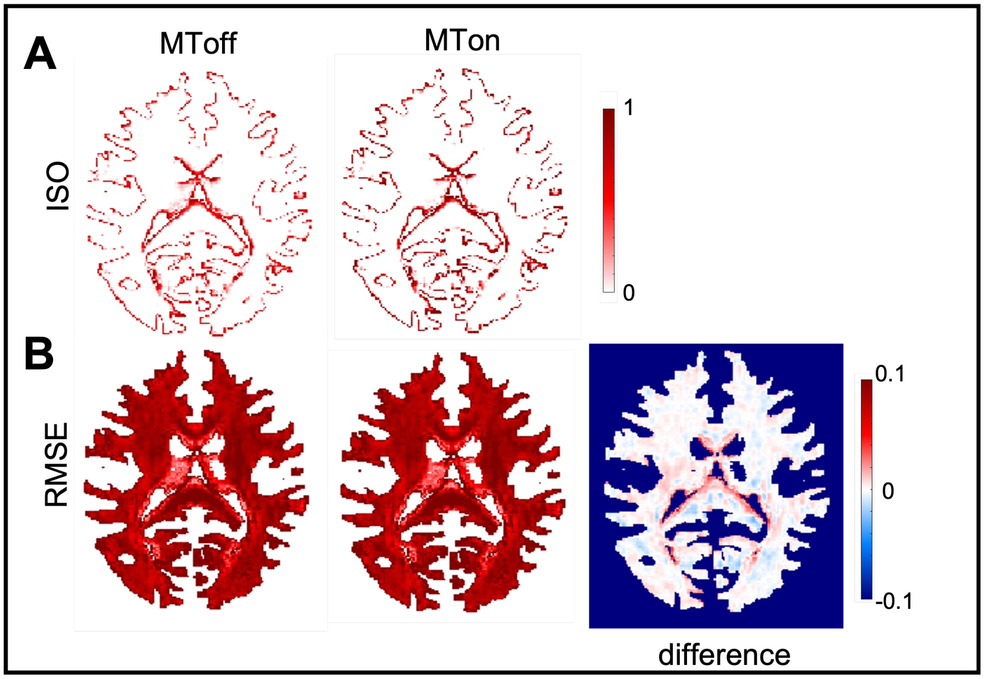

Figure S3: (A) Signal fraction of the 'ball' or ISO compartment for MT_{on} and MT_{off}. (B) The RMSE in MT_{on} and MT_{off} and their difference.

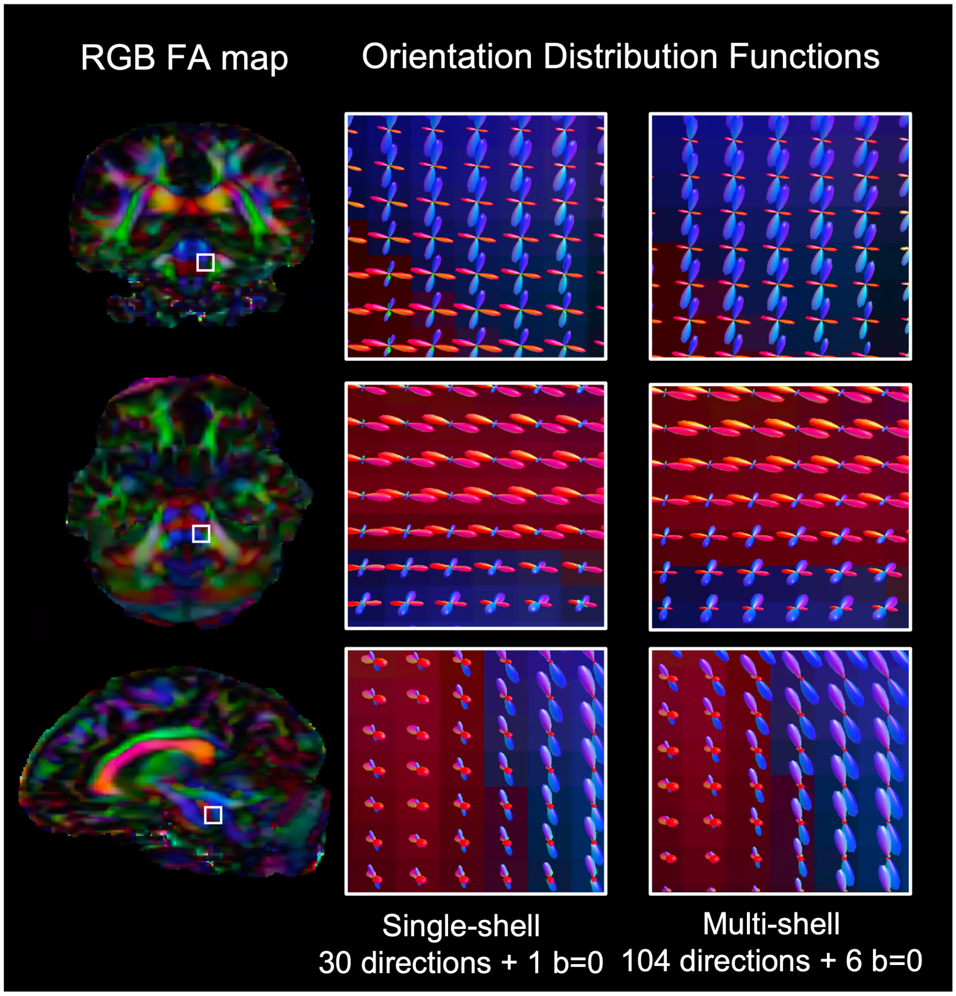

Figure S4: Example of FODs in CST and pontine fiber crossings from single-shell and multi-shell diffusion data
